# Using interpretable decision trees to explore TB-HIV integration under data constraints: a proof-of-concept analysis

**DOI:** 10.3389/ftubr.2026.1853414

**Published:** 2026-06-25

**Authors:** Ntandazo Dlatu, Lindiwe Modest Faye, Mojisola Clara Hosu, Teke Apalata

**Affiliations:** 1 Walter Sisulu Institute for Clinical Governance, Healthcare Administration, School of Public Health, Faculty of Medicine and Health Sciences, Walter Sisulu University, Mthatha, South Africa; 2 Department of Laboratory Medicine and Pathology, Walter Sisulu University, Mthatha, South Africa

**Keywords:** decision tree, exploratory analysis, health systems, HIV, implementation science, integrated care, proof-of-concept study, TB-HIV integration

## Abstract

**Background:**

Strengthening tuberculosis (TB) and HIV service integration remains a major priority in resource-limited settings, where fragmented care contributes to delayed diagnosis, poor treatment continuity, and suboptimal patient outcomes. Evaluating implementation conditions associated with integrated care remains challenging because health system data are often simplified, inconsistently recorded, and insufficiently structured to capture the complexity of real-world service-delivery processes. This study aimed to demonstrate the application of an interpretable decision-tree framework to explore selected implementation and service-delivery conditions associated with TB-HIV integration using a highly constrained secondary dataset.

**Methods:**

A quantitative exploratory proof-of-concept analysis was conducted using a simplified secondary dataset comprising 10 TB-HIV integration implementation cases. Funding, healthcare worker training, patient education, medication access, and infection control were included as binary implementation indicators and entered simultaneously into the decision-tree model. The Gini index was used descriptively to assess node impurity and decision structure. Correlation network analysis was performed to examine patterns of variable alignment, with Pearson correlation coefficients (equivalent to the phi coefficient for binary variables) interpreted solely as descriptive indicators of co-occurrence within the dataset. Given the extremely small sample size and simplified binary structure, all analyses were exploratory and illustrative rather than predictive, inferential, or causal.

**Results:**

Funding emerged as the primary discriminating variable in the decision tree, with all cases reporting increased funding classified as successful (6/6) and all cases without increased funding classified as unsuccessful (4/4). Although all implementation variables were evaluated by the algorithm, no additional variable provided sufficient information gain to generate further branching. Descriptive correlation analysis indicated alignment between funding and TB-HIV integration outcomes (r = 0.78), healthcare worker training (r = 0.76), patient education (r = 0.62), and infection control (r = 0.58). These coefficients are presented solely as descriptive indicators of variable alignment and should not be interpreted as measures of effect size, association strength, or statistical evidence.

**Conclusion:**

This proof-of-concept study demonstrates how interpretable decision-tree approaches can be used to explore implementation conditions associated with TB-HIV integration under severe data constraints. The findings do not identify determinants of integration success but rather illustrate how selected implementation conditions were structured within a constrained analytical framework. The study highlights both the utility and limitations of interpretable analytical approaches when applied to small, dichotomized health systems datasets. Future research should employ larger, more granular, process-sensitive, and contextually rich datasets to support robust evaluation of TB-HIV integration in real-world settings.

## 1 Introduction

Tuberculosis (TB) remains one of the leading infectious causes of mortality worldwide and continues to pose a major public health challenge, particularly in high-burden settings. In 2024, an estimated 10.7 million people developed TB globally and approximately 1.23 million people died from the disease (1). People living with HIV remain at substantially higher risk of developing active TB, particularly in sub-Saharan Africa, where HIV-associated TB continues to contribute significantly to morbidity and mortality (1, 2). Despite improvements in screening and treatment access, gaps in integrated TB-HIV care persist, contributing to delayed diagnosis, fragmented treatment pathways, and poor continuity of care (1). Diagnosing TB in people living with HIV remains particularly challenging because of atypical clinical presentations and reduced sensitivity of conventional diagnostic tools in immunocompromised populations (3–8). Although advances such as Xpert MTB/RIF Ultra have improved diagnostic sensitivity, implementation remains uneven across many resource-limited settings because of infrastructural, organizational, and financial constraints (9–12). Consequently, integrated TB-HIV service delivery has become a key strategy for improving patient outcomes, enhancing continuity of care, and strengthening health system efficiency in high-burden settings (13, 14). TB-HIV service integration aims to coordinate prevention, diagnosis, treatment, and follow-up services across TB and HIV programmes to reduce missed opportunities for care and improve service continuity. However, implementation remains influenced by multiple operational and organizational factors, including workforce capacity, funding availability, infrastructure, service organization, and patient support systems (15). Understanding how these factors interact within healthcare systems remains challenging because implementation processes are often complex, context-dependent, and inadequately represented within routine health information systems. Clinical governance provides a useful conceptual framework for understanding how health systems promote quality, accountability, patient safety, and service effectiveness (16–21). However, these dimensions are rarely captured directly in routinely collected implementation data, particularly in resource-constrained settings where information is frequently recorded as simplified administrative indicators. In the present study, clinical governance is therefore used as an interpretive framework rather than as a directly measured construct. The analysis focuses on selected governance-relevant implementation and service-delivery conditions, namely funding, healthcare worker training, patient education, medication access, and infection control. These variables are considered operational indicators of the implementation environment for TB-HIV integration rather than comprehensive measures of governance performance. A further challenge in evaluating TB-HIV integration is the limited ability of available datasets to capture implementation complexity. Many health system variables are recorded as broad administrative categories that do not adequately reflect implementation fidelity, service coordination, contextual variation, or organizational processes (22–26). As a result, analytical approaches capable of providing transparent insights from constrained datasets may be valuable for supporting methodological exploration and hypothesis generation. Although the literature on TB-HIV integration has expanded substantially, most studies focus on clinical outcomes, implementation barriers, or programme effectiveness. Comparatively less attention has been given to the application of interpretable analytical approaches to examine implementation-related conditions in simplified health systems datasets (26, 27). Furthermore, few studies have explored how machine-learning methods may be used transparently and cautiously when data availability is limited. To address this gap, the present study applies an interpretable decision-tree framework as a proof-of-concept analytical approach. Decision trees were selected because they provide a transparent, visually intuitive representation of how variables are structured within a dataset and how classification decisions are made. Rather than identifying determinants of TB-HIV integration success or developing predictive models, the study demonstrates how selected implementation-related conditions can be explored within a highly simplified secondary dataset collected under real-world data constraints (26, 27).

The study, therefore, aimed to examine how selected governance-relevant implementation and service-delivery conditions were structured in relation to TB-HIV integration outcomes using an interpretable decision-tree framework applied to a simplified secondary dataset.

## 2 Study methods

### 2.1 Study design

This study employed a quantitative exploratory proof-of-concept design to examine how selected implementation and service-delivery conditions were structured in relation to tuberculosis (TB) and HIV service integration outcomes. The study was conceived as a methodological demonstration of how interpretable analytical approaches can be applied to constrained health systems data to support exploration of implementation-related patterns. An interpretable decision-tree framework was selected because of its transparency and visual interpretability when applied to simplified datasets. The analysis focused on five governance-relevant implementation conditions: funding, healthcare worker training, patient education, medication access, and infection control. These variables were treated as operational indicators of the TB-HIV integration environment rather than comprehensive measures of clinical governance or health system performance. The study utilized a secondary dataset comprising 10 TB-HIV integration implementation cases, with all variables operationalized as binary indicators. The sample size reflected the availability of sufficiently complete implementation-level observations within the source dataset. Each observation represented an implementation case rather than an individual patient or healthcare facility. Within this framework, the decision tree was used to explore how selected implementation conditions aligned with TB-HIV integration outcomes and to provide a transparent representation of the resulting classification structure. Given the simplified nature of the dataset, the analysis was intended to generate methodological insights and support hypothesis generation rather than predictive modelling or causal inference. Accordingly, the study prioritized interpretability, methodological transparency, and exploratory analysis to examine implementation-related conditions within a constrained health systems dataset.

## 3 Data collection

This study utilized secondary data derived from a previously published study by Dlatu et al. (28), which examined models of TB-HIV service integration in resource-constrained healthcare settings. The dataset was selected because it contained implementation-related variables relevant to the operational environment of TB-HIV integration, including funding allocation, healthcare worker training, patient education, medication access, and infection control measures. The dataset comprised 10 implementation cases. Each case represented a programmatic or service-delivery instance in which TB and HIV services were operationally coordinated within a specific healthcare setting or implementation environment. These observations reflected variation in implementation conditions across service-delivery contexts rather than individual patients or healthcare facilities. The number of cases available for analysis was determined by the completeness of implementation-level information within the source dataset. Cases with missing data for key implementation variables were excluded from the present analysis. No additional sampling procedures were undertaken.

The outcome variable was classified as successful or unsuccessful TB-HIV integration according to the definitions used in the original study. Successful integration referred to implementation settings in which TB and HIV services were operationally integrated, whereas unsuccessful integration referred to settings in which integration was not achieved. The present study adopted these classifications as reported in the source dataset and did not independently reassess integration status. Consistent with the study’s conceptual framework, the selected variables were interpreted as operational indicators of the implementation environment. Funding was considered an indicator of resource availability, healthcare worker training reflected workforce preparedness, patient education represented patient-facing support, medication access reflected service continuity, and infection control represented clinical and organizational safety practices. All variables were coded as binary indicators (Yes/No) in accordance with the structure of the original dataset. This approach enabled a transparent and interpretable analytical framework suitable for examining patterns within the available data. While these indicators represent important dimensions of the implementation environment, they provide a simplified representation of inherently complex organizational and service-delivery processes. Consequently, the dataset should be interpreted as capturing selected implementation conditions relevant to TB-HIV integration rather than the full complexity of implementation dynamics within health systems.

## 4 Data processing and preprocessing

The dataset comprised 10 TB-HIV integration implementation cases with a binary outcome classification, where successful integration was coded as 1 and unsuccessful integration was coded as 0. Preprocessing procedures included data cleaning, binary variable encoding, and consistency checks to ensure accuracy and internal coherence across observations. All implementation variables, including funding, healthcare worker training, patient education, medication access, and infection control, were retained in their original binary format (Yes/No) as reported in the source dataset. Because the dataset contained only binary variables, no scaling, normalization, or transformation procedures were applied. Maintaining the original coding structure ensured consistency with the source data and preserved the interpretability of the analytical framework. The resulting dataset provided a simplified representation of selected implementation conditions associated with TB-HIV integration. Although this structure facilitated transparent analysis and interpretation, it did not capture the full complexity of implementation processes, organizational dynamics, or contextual variation that may influence service integration outcomes. Consequently, findings derived from the dataset should be interpreted within the context of the available implementation indicators and the scope of the secondary data source.

## 5 Statistical and analytical methods

Data analysis was conducted using R (version 4.2.3) within RStudio (version 2023.03.0 + 386). The primary analytical approach employed an interpretable decision-tree framework to examine how selected implementation and service-delivery conditions were structured in relation to TB-HIV integration outcomes. Entropy-based splitting was used to identify variables providing the greatest information gain at each node, while the Gini index was used descriptively to assess node impurity and split clarity. All implementation variables, funding, healthcare worker training, patient education, medication access, and infection control, were entered simultaneously into the decision-tree model. The resulting tree provided a transparent representation of how these variables aligned with TB-HIV integration outcomes within the available dataset. Variables appearing in the final tree structure represent those that contributed sufficient information gain to generate branching, whereas variables not displayed in the final tree were nevertheless evaluated during model construction. To provide a limited assessment of analytical consistency, leave-one-out cross-validation (LOOCV) was performed. Under this procedure, the model was iteratively trained on nine observations and evaluated on the remaining observations, with each case serving once as the test case. Given the size and structure of the dataset, LOOCV was used as an internal consistency check rather than as a measure of predictive performance or generalizability. To complement the decision-tree analysis, a descriptive correlation network analysis was conducted to examine patterns of alignment among the selected implementation variables. Pearson correlation coefficients, equivalent to the phi coefficient for binary variables, were calculated and interpreted solely as descriptive indicators of variable alignment within the dataset. These coefficients were not intended to represent effect sizes, measures of association strength, statistical evidence, or generalizable relationships. No formal inferential testing was undertaken, and *p*-values and confidence intervals were not emphasized. Consequently, all analytical outputs were interpreted descriptively and used to explore patterns among implementation-related conditions within the available dataset. Following reviewer feedback, the logistic decay model included in earlier manuscript versions was removed because it was not directly aligned with the implementation-focused analytical framework. The final analysis, therefore, focused exclusively on interpretable decision-tree and descriptive correlation network analyses.

## 6 Validation and interpretation

Model validation was considered within the context of the dataset’s structure and intended analytical purpose. Leave-one-out cross-validation (LOOCV) was applied as a limited internal consistency procedure to assess whether the observed decision-tree structure remained consistent across iterative training and testing cycles. However, given the small number of implementation cases and the binary nature of the variables, the results should not be interpreted as evidence of predictive performance, model stability, or generalizability. The decision tree was used primarily as an interpretable representation of how selected implementation conditions were structured in relation to TB-HIV integration outcomes within the available dataset. Accordingly, the analytical outputs are best understood as descriptive representations of variable alignment and methodological illustrations of how interpretable analytical approaches can be applied to constrained implementation data. The findings should therefore be interpreted as exploratory and hypothesis-generating. Their potential relevance for policy, programme implementation, or health systems decision-making requires further evaluation using larger datasets, more granular implementation indicators, longitudinal programme data, and contextually rich health systems information.

## 7 Ethics approval

The original study from which these data were derived received ethical approval from the Biomedical Research Ethics Committee of the Faculty of Medicine and Health Sciences, Walter Sisulu University (Ethics Approval No. 29/2014). Institutional permission to conduct the study was granted by the Eastern Cape Department of Health (Approval No. EC_2016RP27_242), and access to participating healthcare facilities was authorized by the relevant facility managers. The present study used secondary data from an approved research project and was conducted in accordance with the ethical principles of the Declaration of Helsinki and applicable institutional guidelines.

## 8 Results

**Figure 1:**
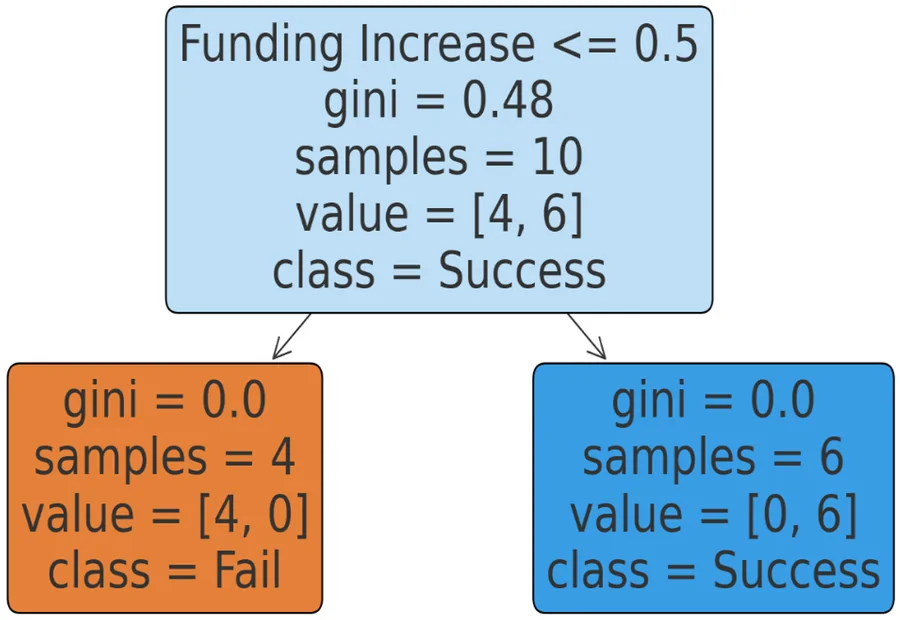
Interpretable decision tree illustrating the alignment of selected implementation variables with TB-HIV integration outcomes. All implementation variables were evaluated during model construction; however, funding generated the greatest information gain and was the only variable selected as a branching node in the final tree structure.

Table 1 summarizes the implementation variables included in the decision-tree analysis and their observed alignment with TB-HIV integration outcomes. All variables, funding, healthcare worker training, patient education, medication access, and infection control, were entered simultaneously into the analytical framework and evaluated during model construction. Funding generated the greatest reduction in node impurity and was therefore selected as the primary split variable. Following this split, terminal nodes achieved complete purity (Gini = 0.0), resulting in no additional branching. Figure 1 presents the decision tree generated from the analysis and illustrates how selected implementation and service-delivery conditions were structured in relation to TB-HIV integration outcomes. The root node labelled “Funding Increase ≤ 0.5,” had a Gini index of 0.48, indicating moderate impurity prior to classification. The dataset comprised 10 implementation cases, including 6 successful and 4 unsuccessful TB-HIV integration outcomes. All cases reporting increased funding (6/6) were classified as successful, whereas all cases without increased funding (4/4) were classified as unsuccessful, resulting in complete purity at the terminal nodes (Gini = 0.0). Although only funding appears in the final tree structure, the remaining variables were evaluated by the algorithm during model construction. Healthcare worker training, patient education, medication access, and infection control did not provide sufficient additional information to generate further branching beyond the initial funding split. Their absence from the final tree should therefore not be interpreted as evidence that they were excluded from the analysis or that they lacked potential relevance in the implementation environment. The simple architecture of the decision tree reflects the structure of the available dataset and should be interpreted as a descriptive representation of how selected implementation conditions aligned within the dataset rather than as evidence of definitive determinants of TB-HIV integration success.

**Table 1:** Variables included in the decision-tree analysis.

Variable	Conceptual Interpretation	Included in Model	Observed Relationship with TB-HIV Integration Outcome^a^	Appeared in Final Tree
Funding	Resource availability and implementation support	Yes	Strong positive alignment	Yes
Healthcare worker training	Workforce preparedness and service capacity	Yes	Positive alignment	No
Patient education	Patient support and engagement	Yes	Positive alignment	No
Medication access	Service continuity and treatment availability	Yes	Positive alignment	No
Infection control	Clinical and organizational safety practices	Yes	Moderate positive alignment	No

a Observed relationships are descriptive and exploratory and should not be interpreted as causal, predictive, or statistically inferential because of the small sample size and binary structure of the dataset.

Figure 1 presents the decision tree generated from the analysis and illustrates how selected implementation and service-delivery conditions were structured in relation to TB-HIV integration outcomes. All implementation variables included in the study funding, healthcare worker training, patient education, medication access, and infection control were entered simultaneously into the decision-tree model. Funding was identified as the only variable providing sufficient information gain to generate a split, resulting in a simple tree structure with a single branching node. The root node, labelled “Funding Increase ≤ 0.5,” had a Gini index of 0.48, indicating moderate impurity prior to classification. The dataset comprised 10 implementation cases, including six successful and four unsuccessful TB-HIV integration outcomes. All cases reporting increased funding (6/6) were classified as successful, whereas all cases without increased funding (4/4) were classified as unsuccessful, resulting in complete purity at the terminal nodes (Gini = 0.0). Although only funding appears in the final tree structure, the remaining variables were evaluated during model construction. Healthcare worker training, patient education, medication access, and infection control did not provide sufficient additional information to generate further branching beyond the initial funding split. Their absence from the final tree should therefore not be interpreted as evidence that they were excluded from the analysis or that they lacked potential relevance in the implementation environment. The simple architecture of the decision tree reflects the structure of the available dataset and should be interpreted as a descriptive representation of how selected implementation conditions aligned within the dataset rather than as evidence of definitive determinants of TB-HIV integration success.

### 8.1 Correlation network analysis

**Figure 2:**
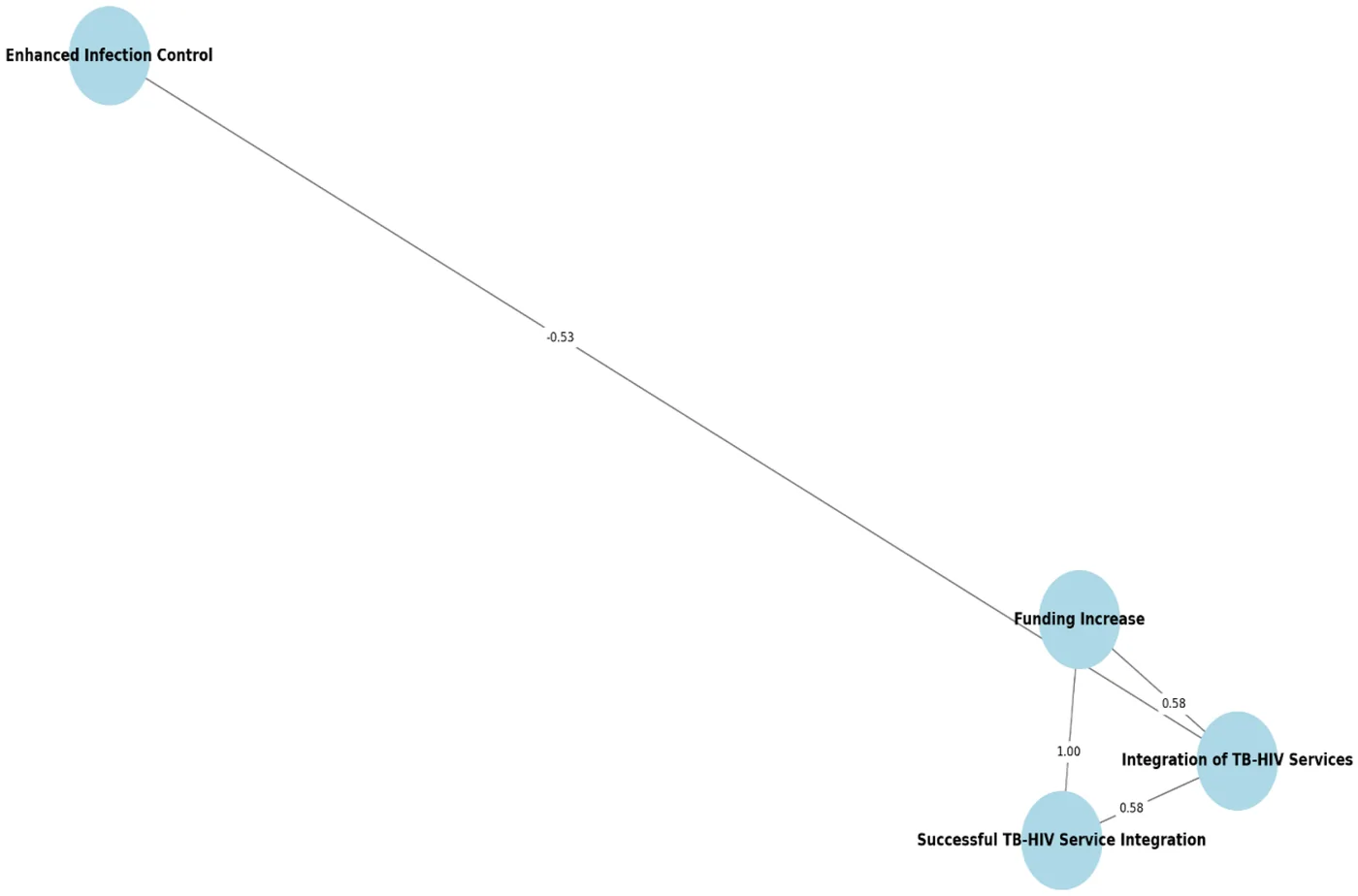
Descriptive correlation network showing patterns of alignment among selected implementation and service-delivery variables associated with TB-HIV integration outcomes.

Figure 2 presents the descriptive correlation network among the implementation and service-delivery variables included in the analysis. The strongest observed correlations were between funding and TB-HIV integration outcomes (r = 0.78) and between healthcare worker training and TB-HIV integration outcomes (r = 0.76). Moderate correlations were observed between funding and healthcare worker training (r = 0.65) and between patient education and TB-HIV integration outcomes (r = 0.62). Weaker correlations were observed between infection control and TB-HIV integration outcomes (r = 0.48) and among several other implementation variables. The correlation network complements the decision-tree analysis by illustrating patterns of alignment among all implementation variables included in the analytical framework, including those that did not appear as branching nodes in the final tree structure. In particular, the network demonstrates that healthcare worker training, patient education, medication access, and infection control remained connected to the broader implementation environment despite not contributing sufficient information gain to generate additional tree branches. Because the correlations were calculated from a small binary dataset, they should be interpreted solely as descriptive indicators of variable alignment within the dataset and not as measures of effect size, association strength, statistical evidence, or generalizable relationships.

## 9 Discussion

This study explored how selected implementation and service-delivery conditions were structured in relation to TB-HIV integration outcomes using an interpretable decision-tree framework applied to a secondary implementation dataset. Within the available data, funding emerged as the primary discriminating variable, with all cases reporting increased funding classified as successful and all cases without increased funding classified as unsuccessful. While this pattern should not be interpreted as evidence that funding alone determines TB-HIV integration success, it highlights the central role that resource availability may play within broader implementation environments. Funding often underpins workforce development, service organization, medication availability, patient support activities, and other operational components required for integrated service delivery. This interpretation is consistent with previous studies demonstrating the importance of financial and organizational support in sustaining integrated TB-HIV programmes (29, 30). The broader pattern of findings is also consistent with existing evidence indicating that healthcare worker training, medication access, patient support, and organizational capacity contribute to the effectiveness of integrated TB-HIV services (30–33). Similarly, the observed alignment involving infection control is consistent with literature emphasizing its importance in protecting both patients and healthcare workers within TB-HIV care environments (34–36). Although these variables did not appear as branching nodes in the final decision tree, the correlation network analysis demonstrated that they remained connected to the broader implementation environment and may contribute to service integration through interactions not captured within the simplified dataset. A key contribution of this study lies in its methodological focus. The findings illustrate how interpretable analytical approaches can be used to explore implementation-related conditions within constrained health systems datasets while maintaining transparency regarding model structure and decision pathways. The decision-tree framework provided a clear visual representation of how variables were organized within the dataset and highlighted the importance of examining all variables included in model construction, rather than focusing solely on those appearing in the final tree structure. The study also underscores the importance of developing more granular and process-sensitive implementation datasets. Many routinely collected health system data sources rely on simplified administrative indicators that may not adequately capture service coordination, implementation fidelity, referral pathways, continuity of care, or organizational context. Emerging methodological work suggests that more structured and activity-based representations of implementation processes may improve analytical validity and provide deeper insights into health system performance and integration dynamics (37, 38). Future research should build on these observations using larger multi-site datasets, longitudinal implementation data, process-level indicators, and context-sensitive measures capable of capturing the complexity of TB-HIV service delivery. Such approaches may help clarify how organizational, clinical, and operational factors interact to influence integration outcomes across diverse healthcare settings. Taken together, the findings support the view that TB-HIV integration is a systems-level process shaped by multiple interrelated implementation conditions rather than by any single factor in isolation. The principal contribution of this study is therefore methodological, demonstrating how interpretable analytical approaches can be applied to explore implementation-related patterns while highlighting the importance of appropriate data structures for health systems analysis.

### 9.1 Policy implications of the study

Although the findings should not be interpreted as direct evidence of programme effectiveness, they offer insights relevant to the implementation of integrated TB-HIV services. Within the available dataset, funding emerged as the variable most strongly aligned with TB-HIV integration outcomes, highlighting the potential importance of resource availability within broader implementation environments. This observation is consistent with implementation research suggesting that financial investment can support workforce preparedness, medication continuity, service coordination, and other operational components required for integrated care delivery. The correlation network analysis further demonstrated that healthcare worker training, medication access, patient education, and infection control were interconnected within the implementation environment. While these relationships should not be interpreted causally, they reinforce the view that TB-HIV integration is influenced by multiple interacting organizational and service-delivery conditions rather than by isolated interventions. A further implication relates to the importance of strengthening health information systems. The study highlights the challenges of evaluating implementation processes using simplified administrative indicators and underscores the value of more structured, process-sensitive, and contextually rich implementation data. Improved data systems may support more robust assessment of service coordination, continuity of care, implementation fidelity, and organizational performance within TB-HIV integration programmes. Accordingly, the principal policy contribution of this study is not the identification of specific intervention priorities but rather the demonstration of the types of implementation insights that may be generated when interpretable analytical approaches are applied to health systems data. Future policy and programme decisions should be informed by larger, more comprehensive datasets capable of capturing the complexity of integrated TB-HIV service delivery.

### 9.2 Study limitations

Several limitations should be considered when interpreting the findings of this study. First, the analysis was based on a small secondary dataset comprising 10 implementation cases, which limited the depth of analysis and the ability to evaluate patterns across diverse implementation contexts. As a result, the observed relationships are highly dependent on the structure of the available data and should not be assumed to reflect broader implementation dynamics. Second, the dataset contained a limited number of implementation-related variables. Important dimensions of TB-HIV integration, including leadership, organizational culture, implementation fidelity, patient-level barriers, service coordination mechanisms, and contextual influences, were not available for analysis. Consequently, the analytical framework captured only a subset of factors potentially relevant to integrated service delivery. Third, all implementation variables were operationalized as binary indicators. While this approach supported transparency and interpretability, it simplified inherently multidimensional implementation processes. Variables such as funding, healthcare worker training, patient education, medication access, and infection control may vary substantially in intensity, quality, timing, and context, dimensions that could not be represented within the available dataset. Fourth, the study was not designed to support predictive modelling, causal inference, or evaluation of programme effectiveness. Rather, the analytical framework was used to explore how selected implementation conditions were structured within the dataset and to demonstrate the application of interpretable analytical approaches under constrained data conditions. Despite these limitations, the study provides a useful methodological illustration of how decision-tree and correlation-based approaches can be applied to explore implementation-related patterns within health systems data. The findings also highlight the importance of developing more granular, process-sensitive, and contextually rich datasets to support future analyses of TB-HIV integration.

## 10 Conclusion

This study applied an interpretable decision-tree framework to examine how selected implementation and service-delivery conditions were structured in relation to TB-HIV integration outcomes. Within the available dataset, funding emerged as the primary discriminating variable, while healthcare worker training, patient education, medication access, and infection control formed part of the broader implementation environment evaluated during model construction. The findings do not establish determinants of TB-HIV integration success or causal relationships among implementation variables. Rather, they demonstrate how interpretable analytical approaches can be used to explore patterns within health systems data and provide transparent representations of variable alignment. The study also highlights the importance of considering all variables included in an analytical framework, including those that may not appear in the final decision-tree structure. More broadly, the findings underscore the need for larger, more granular, and process-sensitive datasets capable of capturing the complexity of integrated service delivery. Future research should incorporate longitudinal implementation data, context-sensitive indicators, and measures of service coordination, continuity of care, and implementation fidelity to support more comprehensive evaluation of TB-HIV integration. Overall, TB-HIV integration is best understood as a systems-level process shaped by interactions among multiple organizational, operational, and contextual factors. The principal contribution of this study lies in demonstrating the potential value of interpretable analytical approaches for exploring implementation-related patterns while highlighting the importance of robust data structures for health systems research.

## Data Availability

The raw data supporting the conclusions of this article will be made available by the authors, without undue reservation.
